# Putrescine-Dependent Re-Localization of TvCP39, a Cysteine Proteinase Involved in *Trichomonas vaginalis* Cytotoxicity

**DOI:** 10.1371/journal.pone.0107293

**Published:** 2014-09-24

**Authors:** Bertha Isabel Carvajal-Gamez, Laura Itzel Quintas-Granados, Rossana Arroyo, Laura Isabel Vázquez-Carrillo, Lucero De los Angeles Ramón-Luing, Eduardo Carrillo-Tapia, María Elizbeth Alvarez-Sánchez

**Affiliations:** 1 Genomic Sciences Postgraduate, Autonomous University of Mexico City (UACM), Mexico City, Mexico; 2 Department of Infectomics and Molecular Pathogenesis, Center for Research and Advanced Studies, IPN, Mexico City, Mexico; University of Quebect at Trois-Rivieres, Canada

## Abstract

Polyamines are involved in the regulation of some *Trichomonas vaginalis* virulence factors such as the transcript, proteolytic activity, and cytotoxicity of TvCP65, a cysteine proteinase (CP) involved in the trichomonal cytotoxicity. In this work, we reported the putrescine effect on TvCP39, other CP that also participate in the trichomonal cytotoxicity. Parasites treated with 1,4-diamino-2-butanone (DAB) (an inhibitor of putrescine biosynthesis), diminished the amount and proteolytic activity of TvCP39 as compared with untreated parasites. Inhibition of putrescine biosynthesis also reduced ∼80% the *tvcp39* mRNA levels according to RT-PCR and qRT-PCR assays. Additionally, actinomycin D-treatment showed that the *tvcp39* mRNA half-life decreased in the absence of putrescine. However, this reduction was restored by exogenous putrescine addition, suggesting that putrescine is necessary for *tvcp39* mRNA stability. TvCP39 was localized in the cytoplasm but, in DAB treated parasites transferred into exogenous putrescine culture media, TvCP39 was re-localized to the nucleus and nuclear periphery of trichomonads. Interestingly, the amount and proteolytic activity of TvCP39 was recovered as well as the *tvcp39* mRNA levels were restored when putrescine exogenous was added to the DAB-treated parasites. In conclusion, our data show that putrescine regulate the TvCP39 expression, protein amount, proteolytic activity, and cellular localization.

## Introduction

Trichomonosis is the most common non-viral sexually transmitted infection (STI) caused by *Trichomonas vaginalis*. This infection mainly affects women, causing vaginitis, cervictis, urethritis, and infertility [1], [2]. It also causes low birth weight infants, preterm delivery [3], and a predisposition to cervical neoplasia [4]. It is also considered as a cofactor in the transmission of the human immunodeficiency virus [5]. According to the genome sequence this parasite contains an expanded degradome of more than 400 peptidases such as metallo, cysteine, serine, threonine, and aspartic peptidases [6]. The *T. vaginalis* cysteine proteinases (CPs) play important roles in trichomonad pathogenesis such as cytoadherence, immune evasion, haemolysis, and cytotoxicity [7]–[12]. The synthesis and proteolytic activity of certain CPs are regulated by environmental factors such as iron, pH, oxidation-reduction capacity, temperature, and polyamines [9],[11],[13]–[15]. The 39 kDa CP (TvCP39), which was found in vaginal washes from patients with trichomonosis and it is localized in the parasite surface, is involved in cytotoxicity to HeLa, DU145 and vaginal epithelial cells (VECs). Interestingly, this CP is *in vivo* and *in vitro* secreted by *T. vaginalis*, and is active in the pH range found in human vagina and prostate [11].

Recently, it has been shown that polyamines are essential nutrient for pathogens that can regulate a variety of trichomonal properties such as cytoadherence and cytotoxicity [14],[16]. A link between trichomonosis infection and polyamines has been suggested by the presence of putrescine in the vaginal fluid of trichomonosis patients [17]–[19]. Quantitative analyses of polyamines in vaginal washes from patients with trichomonosis showed that putrescine and cadaverine are present at high concentrations (0.27 and 0.96 mM, respectively). However, other polyamines as spermine, and spermidine were undetectable [20]. Interestingly, spermine and spermidine are present in the semen at high concentration levels (2.29 and 251 µM, respectively), suggesting that the main contribution of these polyamines is through this fluid [21]. However, the amount of putrescine and other diamines in vaginal secretions were undetectable in patients after get cured [20], suggesting that parasite metabolism is the primary source of putrescine during women infection. Putrescine is synthesized by the ornithine decarboxylase enzyme (ODC), which can be inhibited by polyamine analogues such as 1,4-diamino-2-butanone (DAB) or by 2-difluoromethyl ornithine (DFMO) [19]. Previous studies showed that *T. vaginalis* treated with 20 mM DAB resulted in growth arrest. Additionally, the amount of adhesins involved in trichomonal adherence did not change in DAB-treated parasites; however, an increase in *T. vaginalis* adherence was observed [16]. Interestingly, the addition of 40 mM putrescine to DAB-treated trichomonads was used to rescue growth arrest, and reduced the elevated levels of adherence [16].

Since in TvCP65 is involved in trichomonal cytotoxicity and the expression, protein amount, and proteolytic activity of this CP were reduced in DAB-treated parasites, we suggested that probably exist a relationship between the parasite virulence and polyamines concentration in *T. vaginalis* [14]. However, the polyamines effect on TvCP39 another cysteine protease involved in trichomonal cytotoxicity is still unknown. In this study, we focused on determinate the effect of putrescine on TvCP39 and we found that these cations regulate the *tvcp39* expression, mRNA stability and proteolytic activity, but also the TvCP39 cellular localization.

## Materials and Methods

### 1. *T. vaginalis* culture and inhibition/restoration of putrescine metabolism

Late-logarithmic-phase trophozoites of *T. vaginalis* isolate CNCD147 grown for 24 h in Diamond's trypticase-yeast extract-maltose (TYM) medium pH 6.2 with 10% heat-inactivated horse serum (Gibco) (normal media) at 37°C were used for all assays. The putrescine metabolism inhibition was performed as previously reported [14],[22]. Parasite viability after these treatments was checked by the trypan blue (Sigma) exclusion method [23].

### 2. RNA extraction and cDNA synthesis

Total RNA from 2×10^7^ parasites grown in the absence or presence of 20 mM DAB in TYM medium for 24 h, and DAB-treated parasites transferred into 40 mM exogenous putrescine medium for 30 min at 37°C and into TYM medium (as a control). The RNA was extracted using TRizol reagent (Invitrogen), according to the manufacturer's protocol. Purified RNA was digested with DNase I (Invitrogen) to discard the DNA contaminant, according to the manufacturer's protocol. RNA concentration and purity were determined by measuring absorbance using NanoDrop 2000 (Thermo Scientific); all 260/280 ratios were between 1.8 and 2.1. Then, 1 µg of total RNA was reverse-transcribed using the Superscript II Reverse Transcriptase Kit (Invitrogen), according to the manufacturer's protocol using the oligo-dT (dT_18_) (10 pmol/µl) primer.

### 3. Analysis of *tvcp39* expression by semi-quantitative and quantitative RT-PCR

To validate the expression of *tvcp39* in different putrescine conditions, RT-PCR analysis were performed using 50 ng cDNA from parasites grown in the absence or presence of 20 mM DAB, or DAB-treated parasites transferred into 40 mM exogenous putrescine medium, 10 pmol of each primer pair and 0.25 U of Taq DNA polymerase (Invitrogen). PCR was carried out in a GeneAmp PCR System 9700 thermal cycler (Applied Biosystems Inc., Foster City, CA, USA). Specific primer pairs were designed using Primer3 software version 3.0 (www.primer3.sourceforge.net). We used the following primer pairs to amplify: 110 bp of the *tvcp39* gene (accession number XM_001316379), sense (CP39-FRT) 5′ CAGTATGCTATCACAACAGG 3′ and antisense (CP39-RRT) 5′ CGCCCTGGTGCTTGACAACAT 3′; and 112 bp of the *β-tubulin* gene as reported [24]. The amplified products were analyzed on 2% agarose gels and visualized by ethidium bromide staining. Gene expression densitometry analyses were performed using the Quantity One Software (BioRad). Data from densitometry quantification of the housekeeping gene (*β-tubulin*) were used to normalize the results.

To further support the semi-quantitative data, qRT-PCR was performed using the SYBR Green (QIAGEN) stain, according to the manufacturer's instructions. Specific primers pairs were used: sense CP39-FRT and antisense CP39-RRT. The reaction was carried out in optical 96-well standard plates (Applied Biosystems). PCR was performed with an initial incubation at 94°C for 3 min, followed by 40 cycles at 94°C for 30 s, 60°C for 30 s, and 72°C for 30 s. The reaction was terminated by a final incubation at the dissociation temperatures. The relative quantification of *tvcp39* expression was calculated after the threshold cycle (*C*t) and was normalized with the *C*t of *β-tubulin* (*β-tub*) gene. Furthermore, the expression of *tvcp39* in different putrescine conditions was expressed as normalized Ct values. All reactions including no-template and RT minus controls for each mRNA were run in triplicate. All experimental data were expressed as means ± standard deviation (SD) from three separate biological experiments. The significance of the difference between means was determined by ANOVA with Prisma Firewall 1.53 software. The level of significance was also determined by the Bonferroni multiple comparisons test.

### 4. Actinomycin D Half-Life Experiments

*tvcp39* mRNA stability was monitored in DAB-treated, DAB-putrescine-treated, and untreated parasites using the transcriptional inhibitor actinomycin D (Sigma). Trichomonads were incubated in TYM medium with 50 µg/ml actinomycin D in dimethyl sulfoxide [25] at 37°C. Parasites (2.0×10^7^) were taken at different time-points (0, 1, 3, 6, 8, 12, and 24 h) after transcriptional blockage. Total RNA from trichomonads was extracted by TRIzol, followed by semi-quantitative RT-PCR analysis to detect the presence and stability of *tvcp39* mRNA. The *tvcp39* and *β-tub* mRNA levels were analyzed on ethidium bromide–stained agarose gels and quantified by densitometric analysis with the Quantity One software (BioRad). The *tvcp39* mRNA levels were normalized with the *β-tub* mRNA. The experiment was performed by triplicate and the data were used to calculate the half life. The pixels produced by the *tvcp39* transcript in trichomonads cultured without treatment (t_0_) were defined as 100% for each condition to determine the stability time of *tvcp39* transcript. The experimental *tvcp39* mRNA half-life (the time at which 50% of mRNA molecules remained intact) was determined by the quantity of *tvcp39* mRNA at different times. The theoretical half-life of *tvcp39* mRNA was obtained from the logarithmically transformed best-fit line by linear regression analysis using the decay equation *t½*  =  ln 2/*K*, where *K* corresponds to the decay constant, using the Sigmaplot program.

### 5. Western Blot assays

Cytoplasmic, nuclear and total protein extract obtained from (2×10^7^) DAB-putrescine-treated and untreated parasites were loaded on a 12% polyacrylamide gel with an equivalent of 4×10^5^ parasites/lane. Proteins were transferred to nitrocellulose membranes (BioRad) for 20 min at 20 V using a semi-dry transfer electroblotting system (Trans-blot SD Semi-Dry Transfer Cell, BioRad). The membranes were blocked with 5% skim milk in PBS pH 7.0 –0.05%Tween solution at 4°C for 18 h and subsequently incubated at 4°C overnight with distinct antibodies anti-CP39 (1∶1000 dilution), anti-PCNA (1∶3000) [26], anti-nucleoporin antibody (1∶1000), anti-TveIF-5A antibody (1∶100) [22] or anti-α tubulin (1∶3000)(Zymed Laboratories, South San Francisco, CA) used as a control. After 3 washes with PBS, the peroxidase conjugated secondary antibody (1∶3000) was added to the membrane and incubated at room temperature for 1 h, washed with PBS, developed by the enhanced chemiluminescence ECL Plus Western Blotting Detection System (GE Healthcare), using a Kodak AR film (Kodak, Rochester, NY) exposed for 5 min.

### 6. Indirect immunofluorescence assays

Parasites grown in the presence or absence of 20 mM DAB were fixed using 4% paraformaldehyde for 1 h at 37°C and washed with PBS pH 7.0. Half of the fixed parasites were permeabilized using 1 M HCl for 2 h at room temperature, blocked with 0.2 M glycine for 1 h at 37°C followed by 0.2% fetal bovine serum for 15 min. Then trichomonads were incubated with polyclonal mouse anti-TvCP39 antibody (1∶100 dilution) or preimmune sera (PI) for 18 h at 4°C, washed with PBS, incubated with fluorescein isothiocyanate-conjugated anti-mouse immunoglobulins (1∶90 dilution, Jackson ImmunoResearch) for 40 min at room temperature, washed and mounted with Vectashield-DAPI mounting solution (Vector Lab).

For re-localization assays, parasites grown in the presence of DAB and transferred into 40 mM exogenous putrescine were fixed, permeabilized, and blocked as previously described. Trichomonads were then incubated with polyclonal rabbit anti-TvCP39 antibody (1∶100 dilution) and polyclonal mouse HSP70 antibody (1∶150) for 18 h at 4°C. Parasites were incubated with fluorescein isothiocyanate-conjugated anti-rabbit and tetramethylrhodamine isothiocyanate (TRITC) anti-mouse immunoglobulins (both 1∶90 dilution, Jackson ImmunoResearch) for 1 h at room temperature, and Vectashield-DAPI mounting solution was added. All samples were observed and analyzed using a Leica, DMLS laser-scanning confocal microscopy, and all photographs were taken at the same exposure time.

### 7. Translational blockage by cycloheximide treatment

Translational inhibition in trichomonads cultured in normal and DAB-treated conditions was obtained by adding 10 µg/ml of cycloheximide (Sigma) into the culture medium at time 0 of growth at 37°C [25] and monitored at different time-points (0, 4, 8, 12, and 24). Normal and DAB-treated parasites were transferred into 40 mM putrescine containing medium and incubated at 37°C for extra time (15, 30, and 60 min). After that, total protein extract was obtained from 2×10^7^ parasites by 10% TCA-precipitation as previously described [9]. Solubilized proteins were boiled in sample buffer [27], separated by SDS-PAGE in a 12% polyacrylamide gel, transferred onto nitrocellulose membranes, and western blot assays were performed to detect TvCP39 using the anti-TvCP39 antibody [28]. The anti-tubulin antibody was used as a loading control. Three independent experiments were performed for each time interval, and each measurement was in duplicate.

### 8. Proteinase activity and cell-binding assay

The cell-binding assay to detect proteinases with affinity to the host cell surface was performed as previously described [29]. Parasites (2×10^7^) grown in the absence or presence of DAB, and DAB-treated parasites recovered by exogenous putrescine addition were incubated for 18 h at 4°C with 1×10^6^ fixed HeLa cells. Then trichomonad proteinases bound to the surface of fixed cells were eluted in Laemmli buffer [27] for 20 min at 37°C. Released proteinases were separated on 10% SDS-PAGE gel copolymerized with 2% gelatin. Gels were washed with 10% Triton X-100 for 10 min with gentle agitation and proteinase activation was performed in 100 mM sodium acetate buffer pH 4.5 with 0.1% β-mercaptoethanol for 18 h at 4°C. The gels were further stained with Coomassie Brilliant Blue for a visualization in which clear bands against a dark background indicate proteolytic activity. In addition, we analyzed the proteinase activity of total protein extracts from all conditions as controls. Densitometry analyses of activity bands were performed in triplicate using the software Quantity One version 4.6.3 (BioRad).

Furthermore, the proteolytic activity of the cytoplasmic and nuclear extract was determinate as described above.

## Results

### The putrescine effect on the TvCP39 proteolytic activity

**Figure 1:**
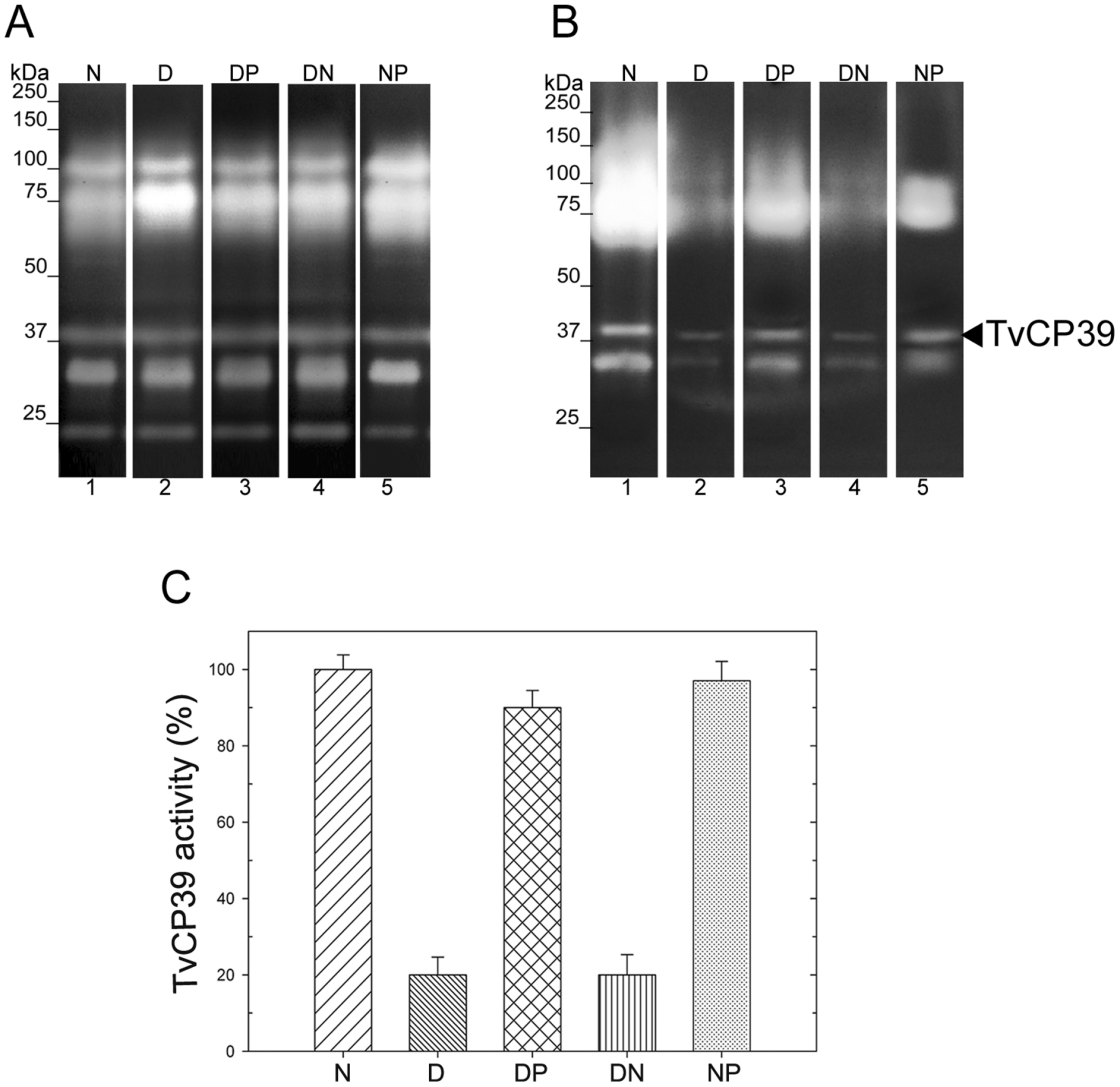
Putrescine effect on the TvCP39 activity from *T. vaginalis*. A) Putrescine effect on the proteolytic activity of *T. vaginalis*. Zimograms using total proteinases from parasites grown in normal media (N)(lane 1), DAB-treated parasites (D)(lane 2), DAB-treated parasites transferred into exogenous putrescine (DP)(lane 3), DAB-treated trichomonads transferred into a normal medium (DN)(lane 4) and parasites grown in normal medium transferred into an exogenous putrescine media (NP)(lane 5). B) Polyamine effect on the proteinases activity bound to HeLa cells. Ligand-proteinases assays using untreated parasites grown in normal medium (N)(lane 1); DAB-treated parasites (D)(lane 2); DAB-treated parasites transferred into exogenous putrescine media (DP)(lane 3), DAB-treated parasites transferred into normal media (DN)(lane 4) and parasites grown in normal media and transferred into an exogenous putrescine media (NP)(lane 5). Arrowhead shows the TvCP30 proteolytic activity. C) Densitometry analyses of TvCP39 proteolytic activity bands from panel B. Bars indicate the average of the intensity of TvCP39 activity bands from three independent ligand-proteinases assays and error bars represent the standard deviations.

The TvCP39 proteolytic activity was analyzed by zymograms. Fig. 1A shows the proteolytic activity of total protein extract from trichomonads grown in normal media (N) (Fig. 1A, lane 1), 20 mM DAB-treated trichomonads (D) (Fig. 1A, lane 2) and DAB-treated parasites and transferred into exogenous putrescine medium (DP) (Fig. 1A, lane 3). In addition the proteinase activity pattern from DAB-treated trichomonad transferred into normal medium (DN) (Fig. 1A, lane 4) and trichomonads grown in normal media and transferred into exogenous putrescine media (NP) (Fig. 1A, lane 5) were used as controls. The zymograms showed that no major changes were observed on total lysates from the DAB-treated parasites (Fig. 1A, lane 2), as compared with untreated control parasites (Fig. 1A, lane 1). These results are consistent with the previously reported [14]. These extracts were also used for cell-binding assays using fixed HeLa cells followed by substrate gel electrophoresis to analyze the proteolytic activity of the TvCP39 bound to HeLa cells (Fig. 1B). The proteolytic activity from TvCP39 bound to the surface of HeLa cells (N) (Fig. 1B, lane 1) was taken as 100% for comparison. Figure 1C showed the densitometric analyses of the TvCP39 proteolytic activity. Interestingly, the proteolytic activity of TvCP39 decreased ∼80% in DAB-treated parasites (D) (Fig. 1B, lane 2 and Fig. 1C). In DAB-treated parasites transferred to exogenous putrescine (DP) (Fig. 1B, lane 3), the TvCP39 activity was ∼90% restored (Fig. 1C) but not in those parasites transferred into a normal media (DN) (20%) (Fig. 1B, lane 4 and Fig. 1C), suggesting that the activity restoration was due to the exogenous putrescine addition. In the control trichomonads grown in normal media transferred into an exogenous putrescine media (NP)(Fig. 1B, lane 5), the TvCP39 activity was similar to that observed in normal-grown parasites (100%)(Fig. 1C).

### TvCP39 transcript levels, protein amount and localization depend on putrescine

**Figure 2:**
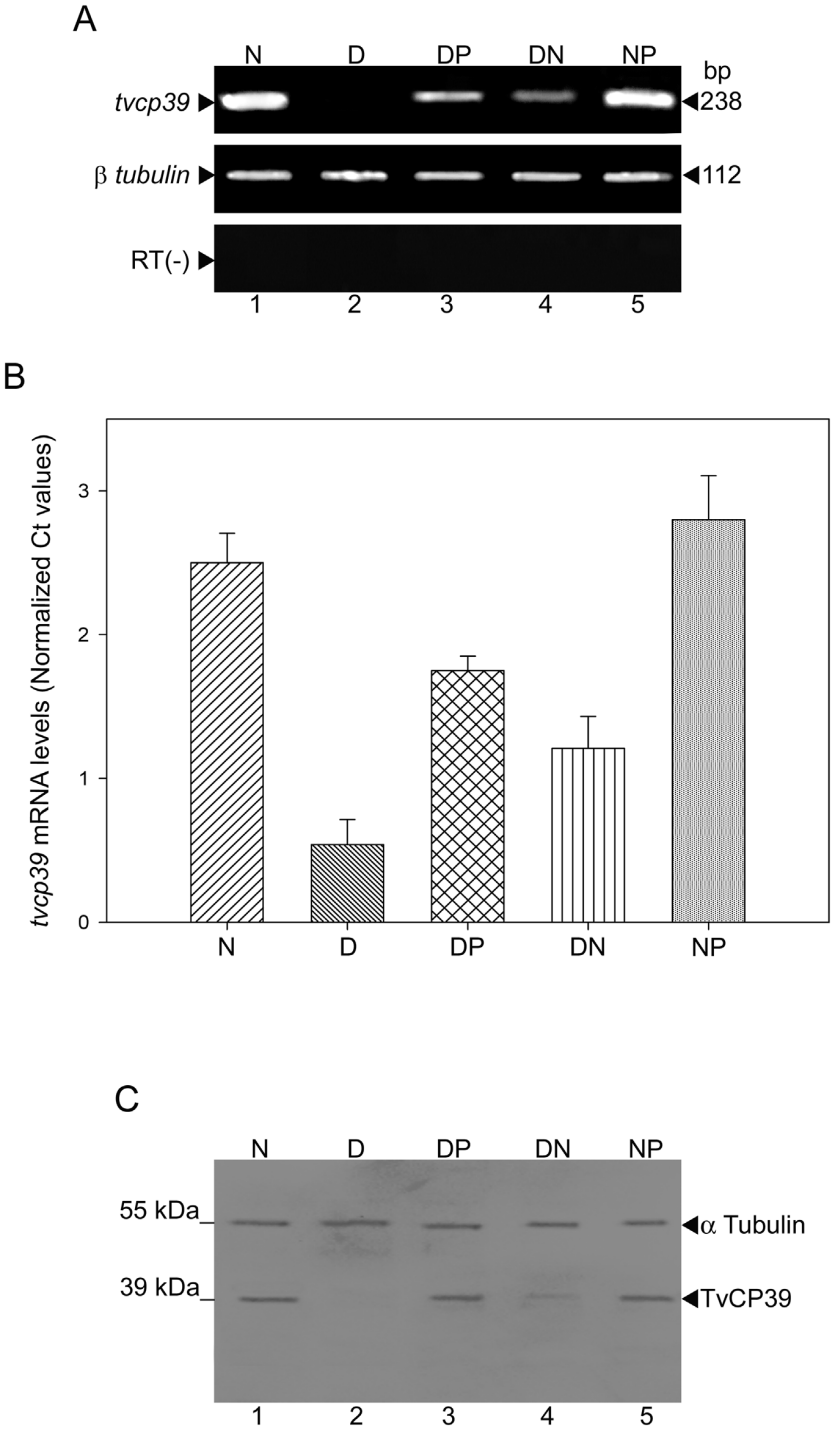
Putrescine effect on TvCP39 transcript and protein. A) Semi-quantitative RT-PCR analysis performed with total RNA from untreated parasites grown in normal medium (N)(lane 1); DAB-treated parasites (D)(lane 2,); DAB-treated trichomonads transferred into 40 mM exogenous putrescine medium (DP)(lane 3); DAB-treated trichomonads transferred to normal medium (DN)(lane 4), and trichomonads grown in normal medium and transferred into 40 mM exogenous putrescine medium (NP)(lane 5) to amplify 238 bp from the *tvcp39*. A 112 pb amplicon from *β-tubulin* was amplify as a loading control. B) qRT-PCR of samples described in A. The Ct levels of *tvcp39* mRNA in trichomonads after DAB treatment (bar D) decreased at 20% but the *tvcp39* mRNA were restored (70%) by adding 40 mM exogenous putrescine to DAB-treated parasites (bar DP). C) Total protein extract from *T. vaginalis* grown in normal media (N)(lane 1); DAB-treated parasites (D)(lane 2); DAB-treated trichomonads transferred into exogenous putrescine media (DP)(lane 3); DAB-treated parasites transferred into normal medium (DN)(lane 4) and trichomonads grown in normal medium transferred to medium with 40 mM exogenous putrescine (NP)(lane 5) were blotted onto nitrocellulose membranes and incubated with anti-TvCP39 and anti-α-tubulin (loading control) antibodies. Arrowheads indicate the immunodetected protein for each antibody employed.

Moreover, to determine whether the *tvcp39* mRNA levels and protein amount correlate with the TvCP39 proteolytic activity, we performed RT-PCR, qRT-PCR and Western blot assays. Consistently, the *tvcp39* mRNA levels decreased in DAB-treated parasites (D)(Fig. 2A, lane 2), and this effect was reverted by the addition of exogenous putrescine (DP)(Fig. 2A, lane 3). In DAB-treated parasites transferred into normal medium a partial recovery of the *tvcp39* mRNA levels was observed (DN)(Fig. 2A, lane 4) and in parasites grown in normal culture medium and transferred into a exogenous putrescine medium, the *tvcp39* mRNA levels (NP)(Fig. 2A, lane 5) were similar to levels observed in parasites grown in normal culture medium (N)(Fig. 2A, lane 1). As a loading control, the 112-bp product from *β-tubulin* was amplified and no changes were observed (Fig. 2A, lanes 1 to 5). Furthermore, qRT-PCR assay showed that the *tvcp39* mRNA expression decreased about 80% (p<0.05) in DAB-treated parasites (Fig 2B, bar D), and addition of exogenous putrescine restored the expression of *tvcp39* mRNA in about 70% (p<0.05)(Fig. 2B, bar DP), compared with trichomonad grown in normal culture medium (Fig. 2B, bar N).

We also analyzed whether the reduction in TvCP39 proteolytic activity correlated with the protein amount by western blot assay using the anti-TvCP39 antibody (1: 6000) [28]. The amount of TvCP39 decreased in DAB-treated parasites (D)(Fig. 2C, lane 2) compared with the amount observed in parasites grown in normal culture media (N)(Fig. 2C, lane 1). However, it was recovered in ∼90 in DAB-treated parasites transferred into exogenous putrescine media (DP)(Fig. 2C, lane 3). In contrast, in DAB-treated parasites transferred into a normal media (DN)(Fig. 2C, lane 4) a partial recovery of TvCP39 amount was observed. In parasites grown in normal culture medium and transferred into a exogenous putrescine medium (NP)(Fig. 2C, lane 5), the TvCP39 amount was similar to the amount observed in parasites grown in normal culture medium (N)(Fig. 2C, lane 1). All this data suggested that the restoration TvCP39 amount and transcript levels were due to the exogenous putrescine addition.

**Figure 3:**
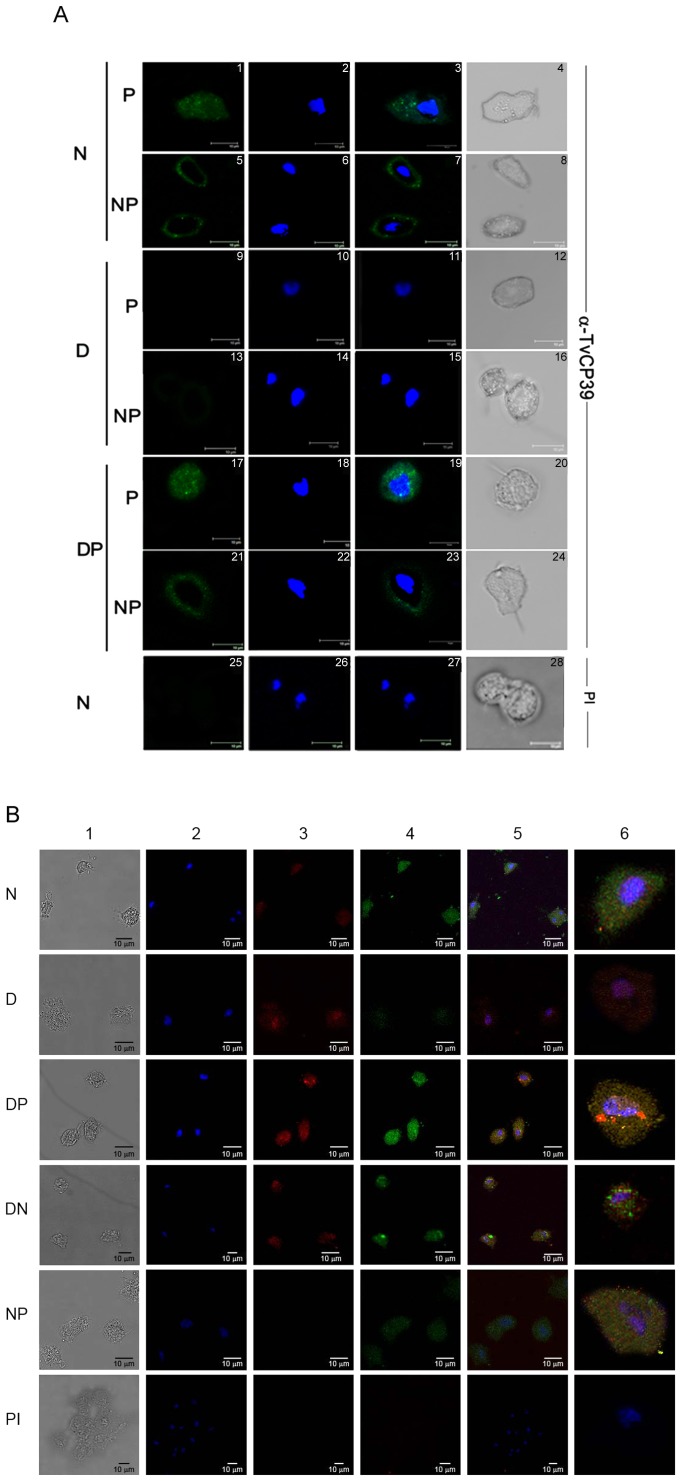
Putrescine effect on TvCP39 localization. A) TvCP39 localization in the polyamine presence. Immunofluorescence analysis of fixed, permeabilized (P; 1–4, 9–12, and 17–20) and Non permeabilized (NP; 5–8, 13–16, and 21,24) parasites untreated (N) (1–8), DAB-treated (D) (9–16), or DAB-treated transferred into exogenous putrescine media (DP) (17–24) incubated with the anti-TvCP39 antibody (1–24) or preimmune sera (PI; 25–28) followed by secondary anti-mouse conjugated to a fluorescein isothiocyanate (Jackson) antibody (1∶90 dilution) and mounted with Vectashield-DAPI. Photographs were taken under laser confocal microscopy (Leica, DMLS). B) Re-localization of TvCP39. Immunofluorescence analyses of fixed and permeabilized parasites that were untreated (Panel N1 to N6) or DAB-treated (Panel D1 to D6), or DAB-treated transferred into exogenous putrescine media (Panel DP1 to DP6), or normal culture parasites that were transferred into exogenous putrescine media (Panel NP1 to NP6). The parasites were incubated with the antibody raised against TvCP39 (green) and anti-HSP70 (red) or with preimmune sera (Panel PI). Nuclei are labeled with DAPI (blue). Photographs were taken under laser confocal microscopy (Leica, DMLS). Scale bar = 10 µm.

Furthermore, we analyzed the putrescine effect over the TvCP39 location by indirect immunofluorescence assays using fixed and permeabilized and non-permeabilized in DAB-treated and untreated parasites. TvCP39 was located in the cytoplasm and at the surface of permeabilized and non-permeabilized parasites, respectively (Fig. 3A, panels 1-8) in normal-grown parasites (N). However, in DAB-treated parasites (D), the TvCP39 fluorescence signal was very low in both types of parasites (Fig. 3A, panels 9–16). Interestingly, the addition of exogenous putrescine (DP) restored the TvCP39 fluorescence signal in the cytoplasm and at the surface of parasites in vesicular forms (Fig. 3A, panels 17–24). Interestingly and unexpectedly, TvCP39 was also observed in the parasite nucleus (Fig. 3A, panels 17–20), suggesting an uncharacterized TvCP39 nuclear function.

In order to confirm the TvCP39 nuclear localization, as a control, we localize HSP70 in the same parasites (Fig. 3B). The TvCP39 was located in the nucleus and nuclear periphery only in DAB-treated parasites transferred into exogenous putrescine media (DP) (Fig. 3B, panels DP1 to DP6) as compared with normal-grown trichomonad (Fig. 3B, panels N1 to N6) and DAB-treated parasites (Fig. 3B, panel D1 to D6), used as controls. HSP70 (red chanel) was localized dispersed in the cytoplasm, nuclear periphery and nucleus in the all conditions (Fig. 3B, panels N3, D3, DP3, DN3, and NP3). Interestingly, in DAB-treated trichomonads that were transferred into exogenous putrescine media, TvCP39 co-localized with HSP70 (Fig. 3B, panel DP6), showed a portion of the protein in the nucleus. These results suggest that TvCP39 is re-localized by the addition of putrescine after DAB treatment.

**Figure 4:**
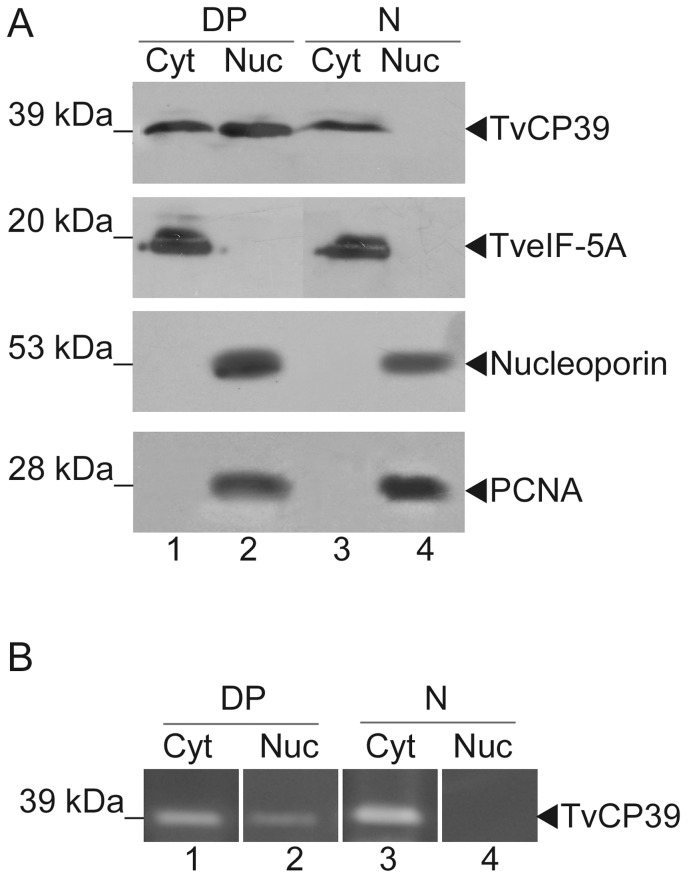
TvCP39 re-localization after DAB treatment and putrescine restoration. A) Cytoplasmic (Cyt) and nuclear (Nuc) protein extract from DAB-treated parasites transferred into exogenous putrescine media (DP) (lanes 1 and 2) and from untreated parasites grown in normal media (N)(lanes 3 and 4) were blotted into a nitrocellulose membrane and incubated with anti-TvCP39, anti-TveIF-5A (control of cytoplasmic protein), anti-nucleoporin (control of nuclear protein) and anti-PCNA (control of nuclear protein) antibodies. Arrowheads show TvCP39 (39 kDa), the TveIF-5A (20 kDa), the nucleoporin (53 kDa), and the PCNA (28 kDa) protein bands. B) Zymograms from Cytoplasmic (Cyt) and nuclear (Nuc) protein extract from DAB-treated parasites transferred into exogenous putrescine media (DP) (lanes 1 and 2) and from untreated parasites grown in normal media (N)(lanes 3 and 4). Arrowhead indicates the TvCP39 proteolytic activity.

Furthermore, cytoplasmic (Cyt) and nuclear (Nuc) protein fractions obtained from parasites grown in the putrescine depleted conditions were analyzed by Western blot assays using the anti-TvCP39 antibody (Fig. 4A).

TvCP39 was localized in the cytoplasmic fraction in normal culture trichomonads (N)(Fig. 4A, panel TvCP39 lane 3) but not in the nuclear fraction (Fig. 4A, panel TvCP39 lane 4). Interestingly, TvCP39 was localized in the nuclear fraction in DAB-treated parasites transferred into exogenous putrescine media (DP)(Fig. 4A, panel TvCP39, lane 2) and in the cytoplasmic fraction (Fig. 4A, panel TvCP39 lane 1). Antibodies anti-TveIF-5A (cytoplasmic protein, 20 kDa), anti-nucleoporin (nuclear pore protein, 53 kDa), and anti-PCNA (proliferating cellular nuclear antigen, 28 kDa) were used as fractionation controls [22],[26]. TveIF-5A was observed in the cytoplasm (Fig. 4A, panel TveIF-5A lanes 1 and 3), consistent with previous report [30]. The nucleoporin protein was immunodetected in the nuclear fraction (Fig. 4A, panel nucleoporin lanes 2 and 4) as previously reported [31]. On the other hand, PCNA has a nuclear localization (Fig. 4A, panel PCNA lanes 2 and 4), this result is in agreement to *Entamoeba histolytica* PCNA protein localization [26]. According to these results, the fractionation was reliable, suggesting that TvCP39 is located in the nucleus only after DAB treatment and restoration with exogenous putrescine addition.

In order to determinate if TvCP39 was an active proteinase when it is localized in the nucleus, we performed zymograms using the cytoplasmic and nuclear fractions described above (Fig. 4B). In normal culture trichomonads (N), we observed the TvCP39 proteolytic activity band in the cytoplasmic (Fig. 4B, lane 3) but not in the nuclear fraction (Fig. 4B, lane 4). Interestingly, in DAB-treated parasites transferred into exogenous putrescine media (DP), we observed a proteolytic activity band corresponding to TvCP39 activity in the nuclear (Fig. 4B, lane 2) and cytoplasmic fractions (Fig. 4B, lane 1).

### The *tvcp39* mRNA stability depends on putrescine

**Figure 5:**
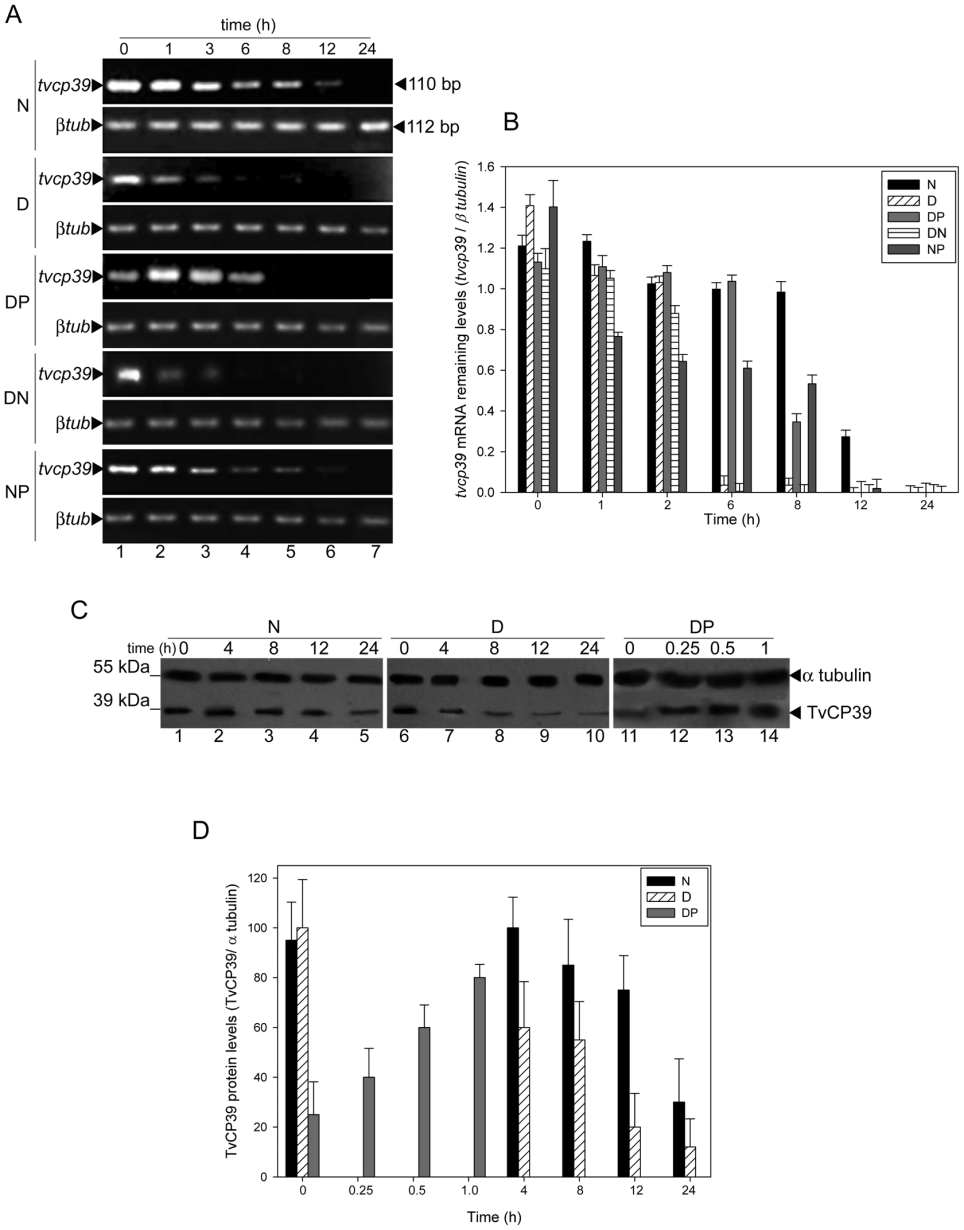
The *tvcp39* mRNA and protein stabilities are regulated by putrescine. A) RNAm levels of *tvcp39* by semi-quantitative RT-PCR analysis using total RNA from parasites treated with actinomycin D and grown in normal culture media (N); or DAB-treated parasites (D); or DAB-treated trichomonads transferred into 40 mM exogenous putrescine medium (DP); or DAB-treated trichomonads transferred to normal medium (DN); or trichomonads grown in normal medium and transferred into 40 mM exogenous putrescine medium (NP). Samples were taken at 0, 1, 3, 6, 8, 12 and 24 h for amplification of 110 pb of *tvcp39* mRNA and 112 bp of *β-tubulin* mRNA (*β-tub)*(loading control). Arrowheads indicate the amplification products obtained. B) Transcriptional blockade using actinomycin D. Trichomonads grown in normal medium (N), DAB-treated trichomonads (D), DAB-treated trichomonads transferred into 40 mM exogenous putrescine medium (DP), DAB-treated trichomonads transferred into normal medium (DN), and trichomonads grown in normal medium and transferred into an exogenous putrescine medium (NP) were treated with actinomycin D. Samples taken at several times (0, 1, 2, 6, 8, 12, and 24 h) were use to amplified the *tvcp39* mRNA which was quantified by densitometric analysis and normalized. Bars represent the mean of each sample and the standard errors were included. C) Blockage of protein synthesis by cycloheximide. Trichomonads were treated with 10 µg of cycloheximide and grown in normal culture media (N); or DAB-treated parasites (D); or DAB-treated trichomonads transferred into 40 mM exogenous putrescine medium (DP). Samples were taken at several times for Western blot analysis using anti-TvCP39 (dilution 1: 1000) and α-tubulin (dilution 1∶100) antibodies. Arrowheads indicate the TvCP39 and α-tubulin proteins. D) Densitometric analysis of the samples described in C. The bands corresponded to TvCP39 were quantified and normalized to α tubulin. Bars represent the mean of three biological triplicates.

Furthermore, we evaluate the putrescine effect over the mRNA stability. In untreated parasites, the *tvcp39* mRNA stability was 12 h after the transcription blockage (Fig. 5A, panel N tvcp39, lanes 1 to 6). In contrast, in DAB-treated parasites, the mRNA stability diminished up to 3 h after transcriptional blockage (Fig. 5, panel D tvcp39, lanes 1 to 3). Interestingly, the *tvcp39* RNAm stability is restored in DAB-treated parasites transferred into putrescine medium (Fig. 5A, panel DP tvcp39, lanes 1 to 4). In DAB-treated parasites transferred into normal medium, no *tvcp39* mRNA stability recovery was observed (Fig. 5A, panel DN tvcp39, lanes 1 to 3). Besides, in parasites grown in normal medium and transferred into exogenous putrescine (Fig. 5A, panel NP tvcp39, lanes 1 to 6), the *tvcp39* stability observed was similar from parasites grown in normal culture media. The *β-tubulin* transcript was used as a loading control (Fig. 5A, panels N βtub, D βtub, DP βtub, DN βtub, and NP βtub) and its stability (>24 h) did not change in all tested conditions.

The *tvcp39* mRNA half-life was estimated to be ∼2.5±0.5 h in parasites grown in normal culture medium (Fig. 5B, N). In DAB-treated parasites, the transcript half-life was 45±10 min (Fig. 5B, D). Interestingly, the *tvcp39* mRNA half-life in DAB-treated parasites transferred into exogenous putrescine media was ∼2.3±0.5 h (Fig. 5B, DP). The DAB-treated parasites transferred into normal medium, and parasites grown in normal medium and transferred into exogenous putrescine medium were used as controls (Fig. 5B, DN and NP).

### Putrescine is necessary for TvCP39 stability

Finally, we compared the effects of actinomycin D and cycloheximide on TvCP39 protein stability in parasites grown in the presence or absence of putrescine using western blot assay (Fig. 5C). In normal culture conditions (N), TvCP39 protein was present up to 12 h (Fig. 5C, lanes 1 to 4); however, at 24 h its amount decreased considerably (Fig. 5C, lane 5). In contrast, in DAB-treated parasites (D) the TvCP39 protein amount started to decrease at 4 h (Fig. 5C, lane 7) and it was continued decreasing until 24 h (Fig. 5C, lanes 8 to 10).

Interestingly, in DAB-treated parasites transferred into exogenous putrescine medium (DP), a TvCP39 amount restoration was observed (Fig. 5C, lanes 11 to 14). These results suggest that putrescine is necessary for TvCP39 protein stability after DAB treatment. No changes were observed in the immunodetection of αtubulin protein (loading control) in all tested conditions (Fig. 5C, lanes 1 to 14). The densitometric analysis (Fig. 5D) performed using biological triplicates are in agreement with the results described above.

## Discussion

*T. vaginalis* contains multiple cysteine proteinases [6], and TvCP65 is well described as a virulence factor of this parasite [9],[14],[32]. Moreover, another CP with a molecular mass of 39 kDa (TvCP39) participates in the cellular damage caused by *T. vaginalis* [28]. TvCP39 specifically binds to host cell surfaces and is immunogenic in patient with trichomonosis [12],[28],[33]. In this work, we show that TvCP39 proteolytic activity was up regulated by exogenous putrescine addition after DAB treatment. Our finding suggest that TvCP39 proteolytic activity might vary during infection, probably by the fluctuations in putrescine concentrations in the vaginal environment [34]. Interestingly, TvCP39 is down regulated by iron [11]; therefore, we suggest that *T. vaginalis* virulence factors, such as TvCP39, are regulated by several environmental host factors.

Our data show that the decreasing in the TvCP39 proteolytic activity correlated with its amount. These results are similar to those reported for TvCP65 [14]. According to our results, putrescine in *T. vaginalis* play important role in the regulation of TvCP39. In other organisms these polycations also regulate basic functions such as replication, transcription, translation, post-translation modifications. In *Plasmodium falciparum* the polyamines depletion arrested the invasion in the early trophozoite stage [35]. In *Leishmania donovani* the ODC activity is necessary for human infections and survival in the host [36]. These data show the important role of the polyamines metabolism in protozoan. DAB acts as an antiparasitic in others protozoan inhibiting the virulence properties [37],[38], but the mechanism is still unknown. The 39 kDa CP is just one of several CP involved in the cellular damage caused by *T. vaginalis*. Others include the TvCP65 that also requires polyamines for expression [14].

TvCP39 is localized in the cytoplasm and parasite membrane [12],[28]. Interestingly, in DAB-treated parasites and after exogenous putrescine addition, TvCP39 was also detected in the nucleus. This re-localization might be related to a novel TvCP39 function, further studies are necessary to elucidate it.

However, nuclear localization of a CP is not unusual for example; EhCP4 of *Entamoeba histolytica* was localized into cytoplasmic vesicles, the nuclear region and perinuclear endoplasmic reticulum [39]. Interestingly, EhCP4 plays a key role in disrupting the colonic epithelial barrier and the innate host immune response during invasion.

Moreover, the mammalian cathepsin L isoform responsible for proteolytic processing of the N-terminal histone 3 (H3) tail, also has a nuclear localization and this proteinase is an active enzyme in the nucleus [40],[41]. This cathepsin L was originally described as a lysosomal protease; however, in the nucleus plays an important role as a chromosomal regulator in the proteolytic processing of the transcriptional factor CDP/Cux and histone H3 [40],[41]. In addition, serpin A3G (SpiA3G), a proteinase that under a pro-inflammatory stimulus macrophages it was relocalized into the nucleolus that co-localizes with cathepsin L, and only the stimulus induce increased nucleolar localization of SpiA3G. Interestingly, the SpiA3g translocation into the nucleolus might be important in host defense against pathogens [42]. The nuclear localization of all these CPs is usually associated with cell cycle or differentiation. The nuclear localization of TvCP39 might be related to an environmental stress caused by putrescine depletion. Although, the nuclear TvCP39 is an active enzyme, the specific role of this proteinase in the nucleus and its transport mechanism remain unknown. Work is in progress to elucidate them.

On the other hand, the TvCP39 protein and *tvcp39* mRNA stability also were affected by putrescine depletion. Moreover, polyamines depletion decreased the mRNA levels, stability and protein amount of TveIF-5A, a polyamine-dependent protein due polyamines are required for the unique posttranslational modification called hypusination [22], suggesting an autoregulatory mechanism in which TveIF-5A modulates the stability of its own transcript [22].

In conclusion, putrescine affects virulence factors of *T. vaginalis*, such as TvCP39. In putrescine absence, the protein and mRNA stability and also the protein amount decreased. However, the putrescine-depletion effect was reverted by the putrescine exogenous addition.

## References

[1] Schwebke JR, Burgess D (2004) Trichomoniasis. Clin Microbiol Rev 17: 794–803. 15489349 10.1128/CMR.17.4.794-803.2004 PMC523559

[2] El-Shazly AM, El-Naggar HM, Soliman M, El-Negeri M, El-Nemr HE, et al (2001) A study on *Trichomoniasis vaginalis* and female infertility. J Egypt Soc Parasitol 31: 545–553. 11478453

[3] Cotch MF, Pastorek II JG, Nugent RP, Hillier SL (1997) *Trichomonas vaginalis* associated with low birth weight and preterm delivery. Sexually Trans Dis 24: 353–360. 10.1097/00007435-199707000-00008 9243743

[4] Viikki M, Pukkala E, Nieminen P, Hakama M (2000) Gynaecological infections as risk determinants of subsequent cervical neoplasia. Acta Oncol 9: 71–75. 10.1080/028418600431003 10752657

[5] Guenthner PC, Secor WE, Dezzutti CS (2005) *Trichomonas vaginalis*-Induced Epithelial Monolayer Disruption and Human Immunodeficiency Virus Type 1 (HIV-1) Replication: Implications for the Sexual Transmission of HIV-1. Infect Immun 73: 4155–4160. 15972505 10.1128/IAI.73.7.4155-4160.2005 PMC1168584

[6] Carlton JM, Hirt RP, Silva JC, Delcher AL, Schatz M, et al (2007) Draft Genome Sequence of the Sexually Transmitted Pathogen *Trichomonas vaginalis* . Science 315: 207–212. 17218520 10.1126/science.1132894 PMC2080659

[7] Neale KA, Alderete JF (1990) Analysis of the proteinases of representative Trichomonas vaginalis isolates. Infect Immun 58: 157–162. 2403530 10.1128/iai.58.1.157-162.1990 PMC258424

[8] Provenzano D, Alderete JF (1995) Analysis of human immunoglobulin-degrading cysteine proteinase of *Trichomonas vaginalis* . Infect Immun 63: 3388–3395. 7642267 10.1128/iai.63.9.3388-3395.1995 PMC173466

[9] Alvarez-Sanchez ME, Avila-Gonzalez L, Becerril-Garcıa C, Fattel-Facenda LV, Ortega-Lopez J, et al (2000) A novel cysteine proteinase (CP65) of *Trichomonas vaginalis* involved in cytotoxicity. Microbial Pathogenesis 28: 193–202. 10764610 10.1006/mpat.1999.0336

[10] Mendoza-Lopez MR, Becerril-Garcia C, Fattel-Facenda LV, Avila-Gonzalez L, Ruiz-Tachiquin ME, et al (2000) CP30, a Cysteine Proteinase Involved in *Trichomonas vaginalis* Cytoadherence. Infect Immun 68: 4907–4912. 10948104 10.1128/iai.68.9.4907-4912.2000 PMC101697

[11] Hernandez-Gutierrez R, Ortega-López J, Arroyo R (2003) A 39-kDa Cysteine Proteinase CP39 from *Trichomonas vaginalis*, Which Is Negatively Affected by Iron May Be Involved in Trichomonal Cytotoxicity. J Euk Microbiol 50: 696–698. 14736224 10.1111/j.1550-7408.2003.tb00692.x

[12] Hernández-Gutiérrez R, Avila-González L, Ortega-López J, Cruz-Talonia F, Gómez-Gutierrez G, et al (2004) *Trichomonas vaginalis*: characterization of a 39-kDa cysteine proteinase found in patient vaginal secretions. Exp Parasitol 107: 125–135. 15363938 10.1016/j.exppara.2004.05.004

[13] Bozner P, Demes P (1990) Proteinases in *Trichomonas vaginalis* and *Tritrichomonas mobilensis* are not exclusively of cysteine type. Parasitology 102. 10.1017/s0031182000060418 1903875

[14] Alvarez-Sanchez ME, Carvajal-Gamez BI, Solano-Gonzalez E, Martınez-Benitez M, Garcia AF, et al (2008) Polyamine depletion down-regulates expression of the *Trichomonas vaginalis* cytotoxic CP65, a 65-kDa cysteine proteinase involved in cellular damage. International J of Biochem and Cell Biol 40: 2442–2451. 10.1016/j.biocel.2008.04.023 18586550

[15] Coombs GH, North MJ (1983) An analysis of the proteinases of *Trichomonas vaginalis* by polyacrylamide gel electrophoresis. Parasitology 86: 1–6. 10.1017/s0031182000057103 6340036

[16] Garcia AF, Benchimol M, Alderete JF (2005) *Trichomonas vaginalis* Polyamine Metabolism Is Linked to Host Cell Adherence and Cytotoxicity. Infection and Immunity 73: 2602–2610. 15845462 10.1128/IAI.73.5.2602-2610.2005 PMC1087355

[17] Reis IA, Martinez MP, Yarlett N, Johnson PJ, Silva-Filho FC, et al (1999) Inhibition of Polyamine Synthesis Arrests Trichomonad Growth and Induces Destruction of Hydrogenosomes. Antimicrob Agents Chemother 43: 1919–1923. 10428913 10.1128/aac.43.8.1919 PMC89391

[18] Yarlett N, Bacchi CJ (1988) Effect of dl-[alpha]-difluoromethylornithine on polyamine synthesis and interconversion in *Trichomonas vaginalis* grown in a semi-defined medium. Mol Biochem Parasitol 31: 1–9. 3141809 10.1016/0166-6851(88)90139-9

[19] Yarlett N, Bacchi CJ (1994) Parasite polyamine metabolism: targets for chemotherapy. Biochem Soc Trans 4: 875–879. 10.1042/bst0220875 7698477

[20] Chen KC, Forsyth PS, Buchanan TM, Holmes KK (1979) Amine content of vaginal fluid from untreated and treated patients with nonspecific vaginitis. The Journal of Clinical Investigation 63: 828–835. 447831 10.1172/JCI109382 PMC372023

[21] Rui H, Gerhardt P, Mevåg B, Thomassen Y, Purvis K (1984) Seminal plasma characteristics during frequent ejaculation. Int J Androl 7: 119–128. 6724722 10.1111/j.1365-2605.1984.tb00767.x

[22] Carvajal-Gamez BI, Arroyo R, Camacho-Nuez M, Lira R, Martínez-Benitez M, et al (2011) Putrescine is required for the expression of eif-5a in *Trichomonas vaginalis* . Mol Biochem Parasitol 180: 8–16. 21801756 10.1016/j.molbiopara.2011.07.003

[23] Alvarez-Sánchez ME, Solano-González E, Yañez-Gómez C, Arroyo R (2007) Negative iron regulation of the CP65 cysteine proteinase cytotoxicity in *Trichomonas vaginalis* . Microbes Infect 9: 1597–1605. 18023389 10.1016/j.micinf.2007.09.011

[24] Leon-Sicairos CR, Leon-Felix J, Arroyo R (2004) tvcp12: a novel *Trichomonas vaginalis* cathepsin L-like cysteine proteinase-encoding gene. Microbiol 150: 1131–1138. 10.1099/mic.0.26927-0 15133072

[25] Lehker MW, Arroyo R, Alderete JF (1991) The regulation by iron of the synthesis of adhesins and cytoadherence levels in the protozoan *Trichomonas vaginalis* . J Exp Med 174: 311–318. 1856625 10.1084/jem.174.2.311 PMC2118921

[26] Cardona-Felix CS, Lara-Gonzalez S, Brieba LG (2011) Structure and biochemical characterization of proliferating cellular nuclear antigen from a parasitic protozoon. Acta Crystallographica Section D 67: 497–505. 10.1107/S0907444911010547 21636889

[27] Laemmli UK (1970) Cleavage of structural proteins during the assembly of the head of bacteriophage T4. Nature 227: 680–685. 5432063 10.1038/227680a0

[28] Ramón-Luing LdlÁ, Rendón-Gandarilla FJ, Puente-Rivera J, Ávila-González L, Arroyo R (2011) Identification and characterization of the immunogenic cytotoxic TvCP39 proteinase gene of Trichomonas vaginalis. The International Journal of Biochemistry & Cell Biology 43: 1500–1511. 21777690 10.1016/j.biocel.2011.07.001

[29] Arroyo R, Alderete JF (1995) Two *Trichomonas vaginalis* surface proteinases bind to host epithelial cells and are related to levels of cytoadherence and cytotoxicity. Arch Med Res 26: 279–285. 8580681

[30] Carvajal-Gamez B, Arroyo R, Lira R, López-Camarillo C, Alvarez-Sánchez ME (2010) Identification of two novel *Trichomonas vaginalis* eif-5a genes. Infection, Genetics and Evolution 10: 284–291. 10.1016/j.meegid.2009.12.008 20060503

[31] Grünwald D, Singer RH, Rout M (2011) Nuclear export dynamics of RNA-protein complexes. Nature 475: 333–341. 21776079 10.1038/nature10318 PMC3154952

[32] Solano-González E, Alvarez-Sánchez ME, Avila-González L, Rodríguez-Vargas VH, Arroyo R, et al (2006) Location of the cell-binding domain of CP65, a 65 kDa cysteine proteinase involved in *Trichomonas vaginalis* cytotoxicity. International J of Biochem and Cell Biol 38: 2114–2127. 10.1016/j.biocel.2006.06.003 16891146

[33] Ramon-Luing LA, Rendon-Gandarilla FJ, Cardenas-Guerra RE, Rodrıguez-Cabrera NA, Ortega-Lopez J, et al (2010) Immunoproteomics of the active degradome to identify biomarkers for *Trichomonas vaginalis* . Proteomics 10: 435–444. 19957290 10.1002/pmic.200900479

[34] Sanderson BE, White E, Baldson MJ (1983) Amine content of vaginal fluid from patients with trichomoniasis and gardnerella associated non-specific vaginitis. Br J Vener Dis 59: 302–305. 6604557 10.1136/sti.59.5.302 PMC1046213

[35] Becker J, Mtwisha L, Crampton B, Stoychev S, van Brummelen A, et al (2010) *Plasmodium falciparum* spermidine synthase inhibition results in unique perturbation-specific effects observed on transcript, protein and metabolite levels. BMC Genomics 11: 235. 20385001 10.1186/1471-2164-11-235 PMC2867828

[36] Boitz JM, Yates PA, Kline C, Gaur U, Wilson ME, et al (2009) *Leishmania donovani* Ornithine Decarboxylase Is Indispensable for Parasite Survival in the Mammalian Host. Infection and Immunity 77: 756–763. 19064633 10.1128/IAI.01236-08 PMC2632046

[37] Calvo-Méndez C, Villagómez-Castro JC, López-Romero E (1993) Ornithine decarboxylase activity in *Entamoeba invadens* . International Journal for Parasitology 23: 847–852. 8314367 10.1016/0020-7519(93)90048-4

[38] Arteaga-Nieto P, Villagómez-Castro JC, Calvo-Méndez C, López-Romero E (1996) Partial purification and characterization of ornithine decarboxylase from *Entamoeba histolytica* . International Journal for Parasitology 26: 253–260. 8786214 10.1016/0020-7519(95)00134-4

[39] He C, Nora GP, Schneider EL, Kerr ID, Hansell E, et al (2010) A Novel *Entamoeba histolytica* Cysteine Proteinase, EhCP4, Is Key for Invasive Amebiasis and a Therapeutic Target. Journal of Biological Chemistry 285: 18516–18527. 20378535 10.1074/jbc.M109.086181 PMC2881777

[40] Goulet B, Baruch A, Moon N-S, Poirier M, Sansregret LL, et al (2004) A Cathepsin L Isoform that Is Devoid of a Signal Peptide Localizes to the Nucleus in S Phase and Processes the CDP/Cux Transcription Factor. Molecular cell 14: 207–219. 15099520 10.1016/s1097-2765(04)00209-6

[41] Duncan EM, Muratore-Schroeder TL, Cook RG, Garcia BA, Shabanowitz J, et al (2008) Cathepsin L Proteolytically Processes Histone H3 During Mouse Embryonic Stem Cell Differentiation. Cell 135: 284–294. 18957203 10.1016/j.cell.2008.09.055 PMC2579750

[42] Konjar Š, Yin F, Bogyo M, Turk B, Kopitar-Jerala N (2010) Increased nucleolar localization of SpiA3G in classically but not alternatively activated macrophages. FEBS Letters 584: 2201–2206. 20338168 10.1016/j.febslet.2010.03.031

